# Atomic-Scale Reaction Mechanism of Impact Ignition in Al/Si/PTFE Reactive Materials

**DOI:** 10.3390/polym18192432

**Published:** 2026-10-06

**Authors:** Liangfei Gong, Lieke Ma, Xiaomalong Pu, Bo Li, Yulian Liu, Ao Mei, Jie Wang

**Affiliations:** 1 State Key Laboratory of Mountain Bridge and Tunnel Engineering, Chongqing Jiaotong University, Chongqing 400074, China; liangfeigong@cqjtu.edu.cn (L.G.);; 2 College of Aerospace Engineering, Chongqing University, Chongqing 400044, China

**Keywords:** polytetrafluoroethylene, reactive materials, micro-ignition mechanism, molecular dynamics

## Abstract

**Highlights:**

- Al/Si/PTFE ternary reactive materials are fabricated by cold-pressing sintering for space debris protection.
- Split Hopkinson pressure bar tests reveal that Al lowers the reaction threshold, and Al_1.2_Si_1_PTFE exhibits the lowest critical temperature and best ignition effect.
- Simulations and differential scanning calorimetry disclose that Al-Si synergy generates abundant hotspots and local temperature rise for optimal energy release.

**Abstract:**

Non-metallic polytetrafluoroethylene (PTFE)-based reactive materials (RMs) exhibit relatively high energy density; however, the low shock sensitivity hinders their widespread application in space debris protection. To develop lightweight, high-energy PTFE-based RMs with superior shock-induced energy-release performance, Al/Si/PTFE ternary RMs were produced by a cold pressing and sintering process with additions of Aluminum (Al) and Silicon (Si) powders. The effect of Al and Si content on the impact ignition and energy release of Al/Si/PTFE was investigated by split Hopkinson pressure bar (SHPB) experiments. High-speed photography shows that the addition of Al powder can effectively lower the reaction threshold of Si/PTFE and enhance its energy release efficiency. The critical reaction temperature threshold of Al_1.2_Si_1_PTFE is lower than that of other Al/Si/PTFE RMs, and its primary and secondary ignition intensities are the highest. The micro-ignition mechanism of the Al/Si/PTFE RMs was revealed by molecular dynamics (MD) simulations and differential scanning calorimetry (DSC) experiments. The synergistic effect of Al and Si particles in Al_1.2_Si_1_PTFE under a shock wave produces a more pronounced local temperature rise and more hotspots, ultimately releasing the greatest extent of reaction.

## 1. Introduction

Polytetrafluoroethylene (PTFE)-based reactive materials (RMs) are inert under normal conditions and react upon impact, holding significant application value in the field of high-efficiency destruction. Currently, research has been primarily focused on the preparation processes, mechanical properties, and damage effects of PTFE-based RMs [1,2,3,4].

As a typical type of metal PTFE-based RMs, Al/PTFE has been widely applied in warhead damage and space debris protection due to its excellent performance under impact loading. Through the investigation of preparation technologies and reaction mechanisms [5,6], the impact-induced reaction performance of PTFE-based RMs has been mainly improved from three aspects: particle size regulation, material structure optimization, and addition of small amounts of inert particles or reducing agents [7,8,9,10,11]. Wang et al. [12] established laser ignition experiments on Al/PTFE and pointed out that Al particle size and content have a significant influence on the energy release behavior. The heat release processes of Al/PTFE (mass ratio: 26.5%/73.5%) and core–shell structured Al/PTFE energetic powders in a confined environment were measured by Tang et al. [13]. A significant increase in energy release during the combustion reaction was found in the core–shell Al/PTFE RMs. Song et al. [14] prepared two types of Al/PTFE RMs by adding aluminum honeycomb as a structure (Type 1: Al/PTFE/aluminum honeycomb (20.5 wt%/73.5 wt%/6 wt%); Type 2: Al/PTFE/aluminum honeycomb (24.9 wt%/69.1 wt%/6 wt%)), and found that the aluminum honeycomb-reinforced Al/PTFE RMs display a distinct strain rate effect. Therefore, it can be concluded that both the microstructure of RMs and macro-scale reinforcing structures can promote their reaction performance. In addition, the ballistic experiments of W/Al/PTFE RMs was carried out using a quasi-sealed test chamber, and it was found that under a certain impact velocity, the reaction efficiency is determined by the W content [15]. The Al/PTFE/NC composite materials produced by electrostatic spray technology can achieve more effective combustion [16]. The combustion tests revealed that such an NC binder overcomes the limitations of the natural aluminum oxide shell on the Al surface and the inefficient mass/heat transfer between Al and PTFE. Anaerobic reaction kinetics of thermites found that the addition of Si in the PTFE/Al system can effectively regulate and enhance the energy performance of thermite [17]. A study on energy release behavior of five types of RMs (PTFE/Al, PTFE/Si, PTFE/Al/B, PTFE/Al/Si, and PTFE/Al/Cu) via a drop hammer system demonstrated that PTFE/Al/Si exhibits a strong energy-releasing capacity, making it a promising new option for reactive fragments [18]. However, not all combustion additives are more effective. Song et al. [19] introduced KMnO_4_ and TiH_2_ into classical metal/fluorine-containing reactive materials, and found that the reaction performance of the composite RMs first increases and then decreases with the increase of KMnO_4_ content.

Compared with the typical Al/PTFE RMs, Ti/PTFE RMs have gradually entered the researchers’ field of vision due to their better energy release potential and the same lightweight characteristics. The static and dynamic compressive experiments of Ti/PTFE RMs confirmed that the introduction of Ti powder can improve the material strength under dynamic loading [20]. The dynamic compressive properties and impact-induced reaction behavior of Ti/W/PTFE RMs with different W contents were investigated by split Hopkinson pressure bar (SHPB) and high-speed photography [21]. Results indicated that W power can enhance the inertness of Ti/W/PTFE RMs; that is, the impact ignition reaction threshold is increased with the W content. He et al. [22] compared the damage effects of Ti/Al/PTFE composite jets (PTFE/Al as additives) and Ti jets on multi-spaced targets, and found that the Ti/Al/PTFE composite jets exhibit superior impact damage performance. Therefore, it can be shown that adding a small amount of a third particulate material to metal/fluoropolymer-based systems or incorporating a small amount of RMs into metals can both affect their impact-induced reaction and energy release. However, research on how their contents influence the impact-induced reaction and energy release remains insufficiently limited.

Currently, research on the mechanical properties and reaction performance of non-metallic PTFE-based RMs under impact loading is relatively scarce compared to that of metal-PTFE-based RMs. Existing studies have mainly focused on Si/PTFE, involving preliminary explorations of its gradation relationship, material structure types, mechanical properties, and the influence of adding a small amount of Al on the material’s mechanical and reaction properties. Zhuang et al. [23] found that the relevant ratio has a significant effect on the reactivity of core–shell structured Si/PTFE RMs, and the improved adhesion between reactants induced by the core–shell structure can greatly enhance their energy release performance. Zhang et al. [24] mixed three types of doped silicon (n-Si, p-Si, and i-Si) with PTFE powder to form three Si/PTFE RMs. TG and DSC results have shown that the heat release of p-Si/PTFE is approximately half of that of i-Si/PTFE or n-Si/PTFE, and the critical reaction temperature of p-Si/PTFE and n-Si/PTFE is lower than that of i-Si/PTFE. In addition, some studies have focused on using non-metallic particles only as additives in metal/PTFE-based RMs to investigate their effects on static/dynamic mechanical properties and combustion performance. Ding et al. [25] established a Johnson–Cook constitutive model for PTFE/Al/Si (4:1) considering strain hardening, strain rate hardening, and thermal softening effects. Therefore, it can be seen that the current research on non-metallic/PTFE-based systems is relatively limited, especially regarding their impact-induced reaction performance.

In summary, non-metallic PTFE-based RMs possess the characteristics of high energy density, low density, and low shock sensitivity, while metal/PTFE RMs exhibit advantages of low reaction threshold and high reaction efficiency—particularly Al/PTFE, which has a relatively high reaction rate. Therefore, inspired by the multi-component synergy design concept of multi-principal element medium-entropy alloys, this paper employs PTFE as the matrix and constructs a multiphase composite energetic material by introducing metallic Al and nonmetallic Si particles with different mass fractions (rather than merely a small amount as modifiers), thereby achieving the synergistic improvement of material strengthening and reaction activity. Firstly, SHPB and quasi-closed shock experiments will be conducted to characterize the impact-induced energy release of the Al/Si/PTFE RMs with various Al contents. Secondly, DSC will be employed to determine the reaction onset temperatures of Al/PTFE, Si/PTFE, and Al/Si/PTFE. Finally, the multi-scale simulation method (MSST) proposed by Reed et al. [26] will be adopted to investigate the advantages of the ternary Al/Si/PTFE RMs over their binary counterparts at the microscale level. The evaluation of the overall reaction performance and mechanism of ternary RMs is expected to fully use the high energy density of non-metallic/PTFE RMs and enhance their reaction rate.

## 2. Materials and Methods

### 2.1. RMs Sample

**Figure 1:**
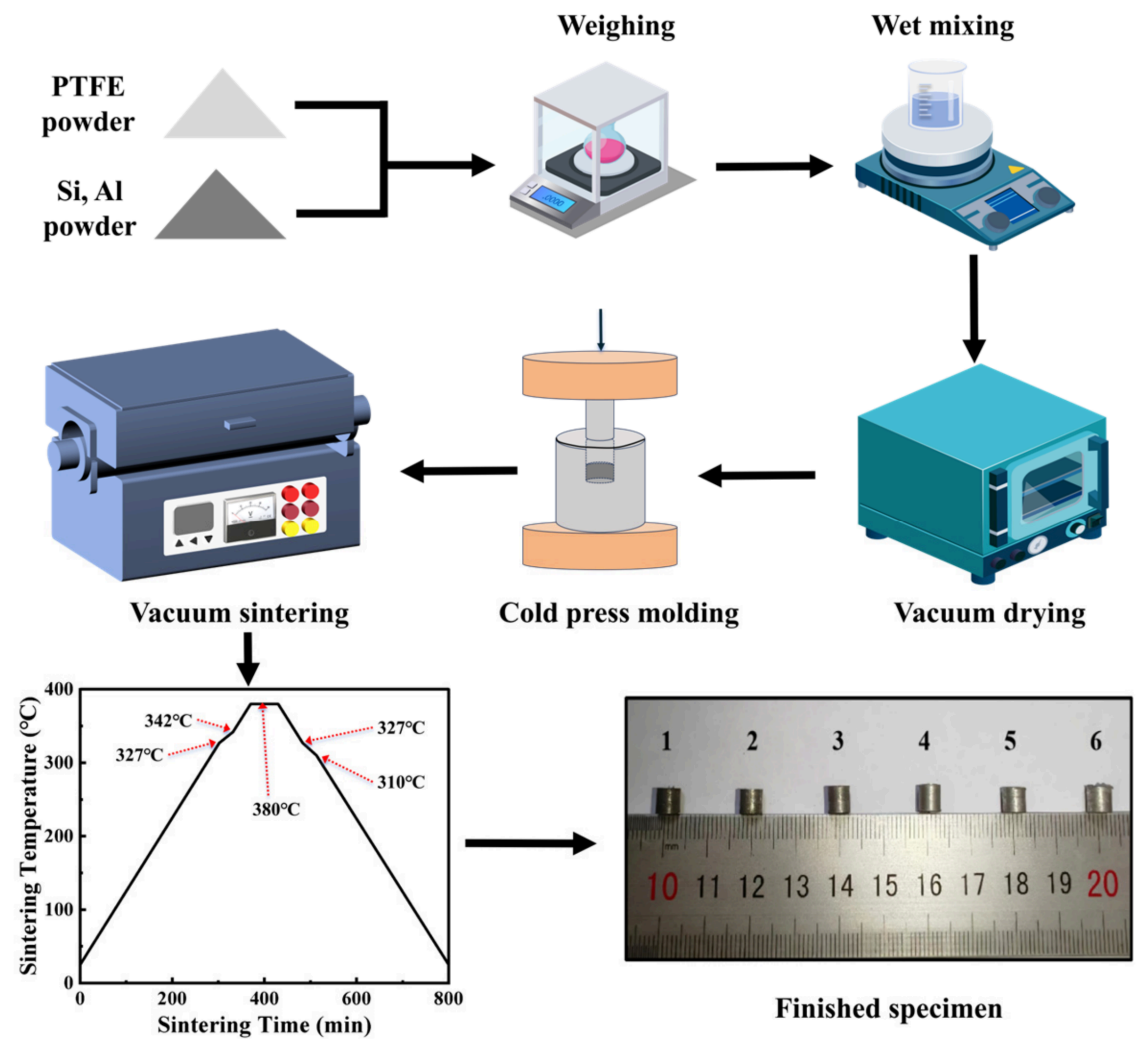
The material preparation process and the specimens prepared.

The samples of RMs were prepared by cold pressing and sintering. The particle sizes of the powders used in this study are as follows: PTFE powder ranging from 20 to 50 μm, Al powder with a size of 1.67 μm, and Si powder with a size of 1 μm. The preparation process and final test specimens are shown in Figure 1. First, each powder was accurately weighed according to the zero-oxygen-balanced ratio by using a precision electronic balance, and then was placed in a beaker. Absolute ethanol was then poured into the powder mixture, which was stirred using a magnetic heating stirrer for 30 min to ensure that the Al and Si powders were uniformly dispersed within the PTFE matrix. The wet-mixed powder was then poured into a baking tray and dried in a vacuum drying oven at 60 °C for 6 h. Subsequently, the dried particle mixture was placed into a cylindrical pressing mold. Cold compaction molding was performed under a pressure of 200 MPa; the pressure was increased to 3926 N at a rate of 5 N/s, and held for 5 min. The compacted samples were sintered in a vacuum/atmosphere tube furnace following a preset sintering temperature profile (Heated to 380 °C at a rate of 50 °C/h and held at this temperature for 6 h. then cooled to 310 °C at a rate of 50 °C/h and held for 4 h. Finally cooled to ambient temperature at a rate of 50 °C/h. After polishing, six types of Al/Si/PTFE ternary RMs having various mass ratios were obtained, all with a size of Φ5×5 mm. The color of the specimens gradually changed from the steel gray of Si to the silvery white of Al as the content of Al in RMs increased. The actual density, theoretical maximum density (TMD), and relative density of six Al/Si/PTFE ternary RMs are presented in Table 1. It can be observed that the relative density of all samples exceeds 96%.

**Figure 2:**
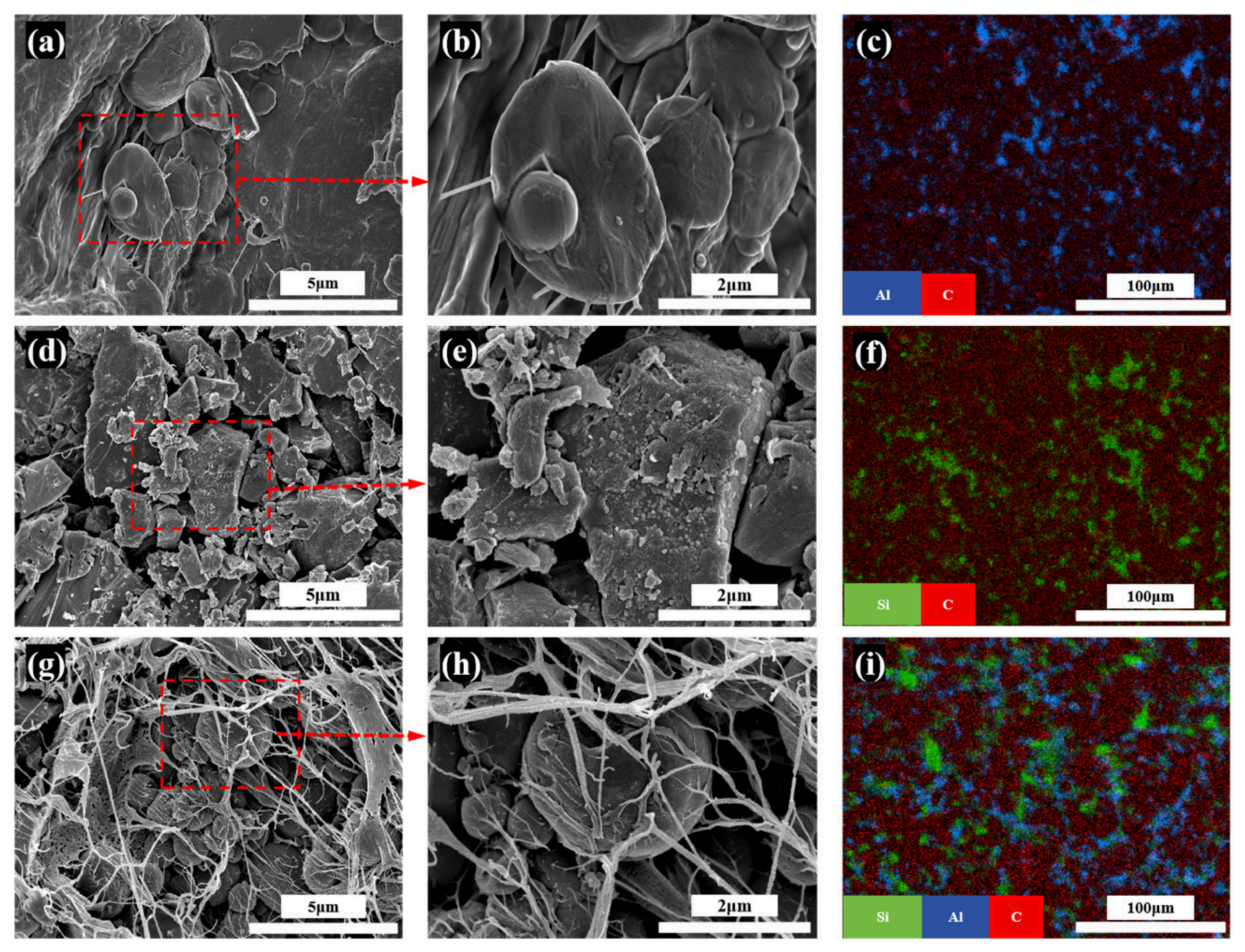
SEM morphologies and EDS mappings of three RMs: (**a**) SEM (Al/PTFE); (**b**) local magnified morphology of Al/PTFE; (**c**) EDS (Al/PTFE)blue; (**d**) SEM (Si/PTFE); (**e**) local magnified morphology of Si/PTFE; (**f**) EDS (Si/PTFE); (**g**) SEM (Al_1.2_Si_1_PTFE); (**h**) local magnified morphology of Al_1.2_Si_1_PTFE; (**i**) EDS (Al_1.2_Si_1_PTFE). The blue, green, and red regions represent Al, Si, and C, respectively.

To obtain the microscopic characteristics of Al/Si/PTFE ternary RMs of various mass ratios, the cold field emission scanning electron microscopy (SEM) and energy dispersive spectroscopy (EDS) were employed (Center for Electron Microscopy, Chongqing University, China). The SEM, by using a focused and narrow beam of high-energy electrons to scan samples, was able to observe the microscopic morphology of RMs’ surfaces. The EDS can analyze the micro-region composition, primarily detecting the types and content of elements of RMs. It can be seen from Figure 2a,b that the spherical Al particles are approximately 1 μm in size, a value that aligns well with the experimental particle sizes. The flaky and blocky Si particles are clearly observed to be distributed on the PTFE matrix from Figure 2d,e, which is consistent with the material sizes used in the experiment. After sintering, the PTFE forms a continuous matrix with pores. When pressure is applied to these pores, stress concentration can initiate cracks that accelerate material failure. In the EDS diagrams, the red regions represent the C element, indicating PTFE distribution. The blue regions represent the Al element, and the green regions represent the Si element. From Figure 2c, it is evident that there is always some PTFE around the Al particles in each region, indicating that the Al particles are uniformly distributed within the PTFE. The Si particles are also uniformly dispersed in the PTFE matrix, as shown in Figure 2f.

Based on the stoichiometric zero-oxygen reactions between Al and Si with PTFE, respectively, the abbreviation Al_1.2_Si_1_PTFE only denotes the mass ratio of Al to Si (i.e., Sample 4#). In Al_1.2_Si_1_PTFE (Figure 2i), Al particles and Si particles are uniformly dispersed in the PTFE matrix. Therefore, the influence of uneven particle distribution on the reaction performance of RMs can be avoided to a certain extent in the prepared specimens.

### 2.2. DSC Analysis

**Figure 3:**
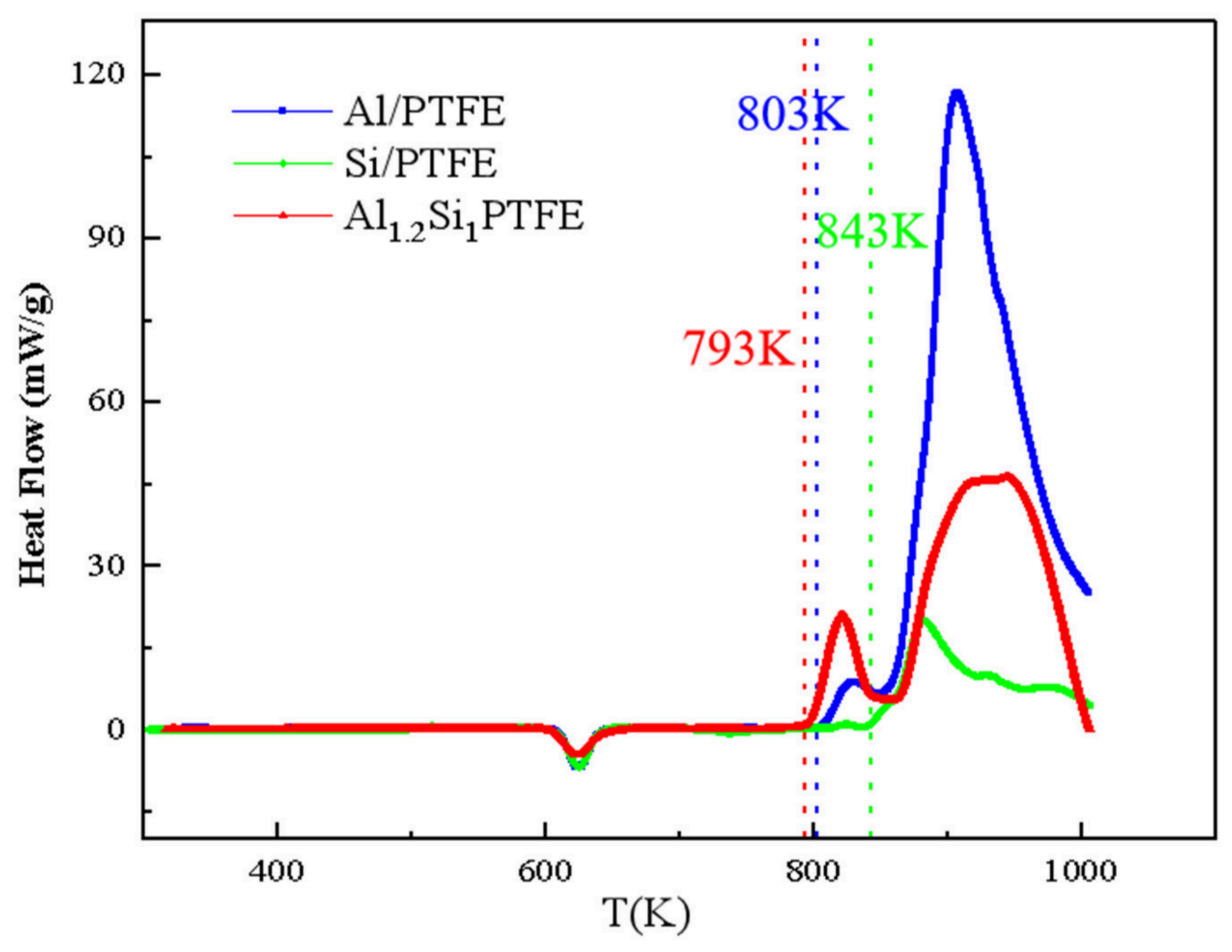
DSC heating test results of three RMs (Al/PTFE; Si/PTFE; Al_1.2_Si_1_PTFE).

To compare the critical reaction-onset temperatures of Al/Si/PTFE, Al/PTFE, and Si/PTFE composites, DSC (SC-DSC3600) was employed to analyze the thermal behavior. Under an argon atmosphere at a flow rate of 100 mL/min, the reactive mixtures were heated from 30 °C to 750 °C at a rate of 10 °C/min, and the heat-flow changes of Al_1.2_Si_1_PTFE, Al/PTFE, and Si/PTFE were recorded as a function of temperature. The experimental results, as shown in Figure 3, demonstrate that all three materials exhibit a distinct endothermic peak around 603 K, caused by PTFE melting. As the temperature rose, Al_1.2_Si_1_PTFE started an exothermic reaction at 793 K, while Al/PTFE started at 803 K and Si/PTFE at 843 K, respectively, indicating that Al_1.2_Si_1_PTFE has the lowest ignition temperature.

### 2.3. Split Hopkinson Pressure Bar Testing

**Figure 4:**
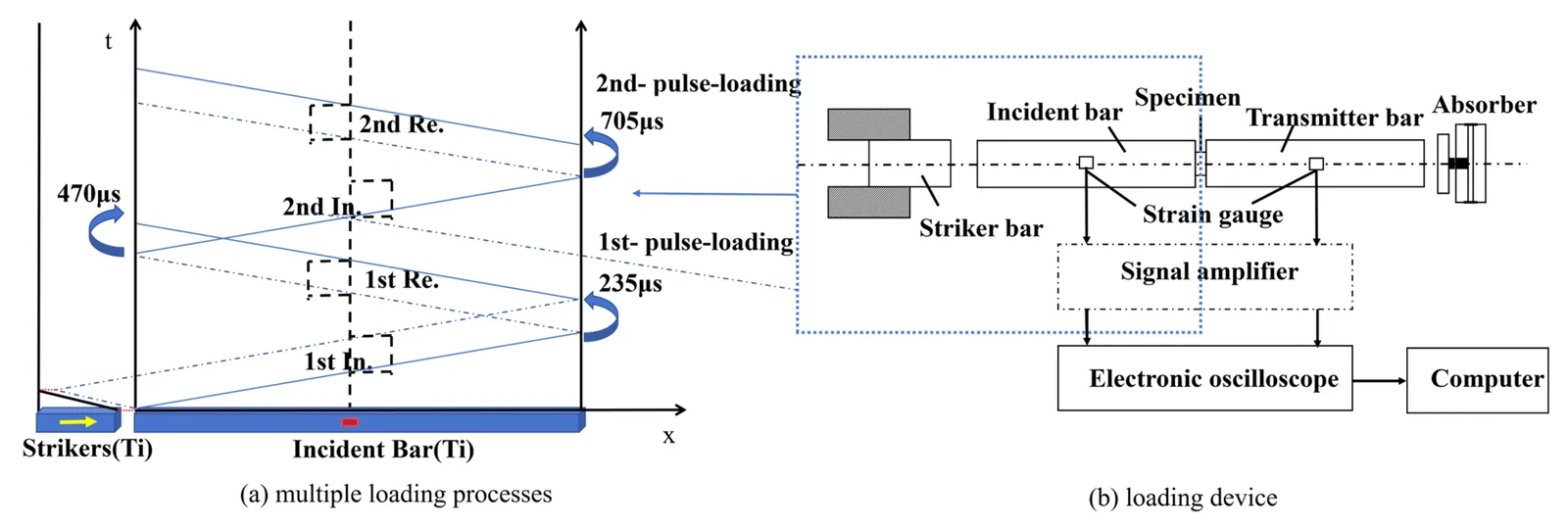
SHPB device and loading diagram.

To explore the ignition reaction characteristics of Al/Si/PTFE ternary RMs, a SHPB with a diameter of 14 mm was utilized. The input bar, the transmission bar with a length of 1200 mm, and the bullet with a length of 300 mm are all fabricated from TC_4_ titanium alloy, which has a sound velocity (*C*_0_) of 5100 m/s. By adjusting the gas chamber pressure (bullet velocity), the strain rate applied to the specimen was controlled. A high-speed camera with a frame rate of 9000 fps and a maximum temporal resolution of approximately 111 μs was used to synchronously record the reaction process of the material, as depicted in Figure 4b. During the experiment, the specimen was subjected to multiple loadings. As shown in Figure 4a, the bullet is driven by the high-pressure gas chamber, generating a stress wave that propagates to the end of the incident bar and delivers the first loading to the specimen. Simultaneously, the reflected stress wave forms another compressive wave at the beginning of the incident bar, which propagates to the end and applies a second loading to the specimen. The interval between the two loadings is 470 μs.

### 2.4. Quasi-Sealed Impact Testing

**Figure 5:**
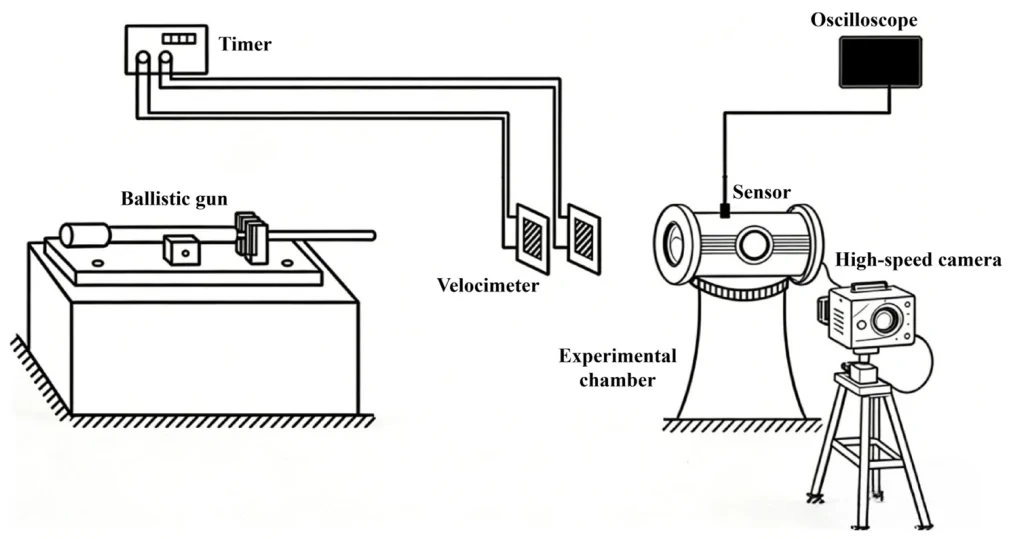
Quasi-sealed impact experimental system.

To quantitatively compare the impact reaction characteristics of Al/Si/PTFE ternary reactive materials under high strain rates, impact experiments were conducted using a quasi-sealed system, as illustrated in Figure 5. The experimental setup consists of a one-stage light-gas gun, a velocimeter, a quasi-closed chamber, and a high-speed camera. When the energetic projectile (Φ5 × 5 mm) impacts a steel plate with a diameter of 12 cm inside the chamber, the overpressure signal generated by the reaction is transmitted from the pressure sensor to an oscilloscope. Meanwhile, the transient impact reaction phenomena of the material at different moments are recorded by the high-speed camera. In this work, only Al/PTFE and Al/Si/PTFE RMs with the optimal mass ratio were compared, with impact velocities ranging from 650 m/s to 750 m/s.

## 3. Theory

To reveal the impact ignition mechanism of Al/Si/PTFE ternary RMs with different Al/Si mass ratios at the microscopic scale, the multiscale shock technique (MSST) method proposed by Reed [26] was applied in LAMMPS. This method addresses the limitations of traditional simulation approaches in handling high-strain-rate problems and offers higher computational efficiency. Its core method is to dynamically adjust the simulation box size and particle velocities to efficiently and stably generate a steady-state planar shock wave, precisely controlling its propagation so that the final state of the simulated system satisfies the Rankine–Hugoniot conservation relations, which are depicted in Equations (1)–(3).

(1) $$\rho_{0}(U_{s}-U_{0})=\rho (U_{s}-U_{p})$$

(2) $$P-P_{0}=\rho_{0}(U_{s}-U_{0})(U_{p}-U_{0})$$

(3) $$e-e_{0}=\frac{1}{2}\left(P+P_{0}\right)\left(v_{0}-v\right)$$

where *U*_p_ is the particle velocity of the material in the laboratory frame, and *U*_s_ is the shock wave velocity. *ρ* is the specific density, *e* is the energy per unit mass, and *P* is the pressure in the direction of shock propagation. *v* is the volume occupied by a unit mass. Variables with subscript 0 are the values before the shock wave. The Hugoniot relations of every substance in RMs are compared with experimental data to verify the fidelity of the potential functions. Then, shock loading was applied to Al/Si/PTFE RMs of various mass ratios, and the thermomechanical responses under impact conditions were analyzed based on shear strain and temperature.

### 3.1. Model and Simulation Settings

**Figure 6:**
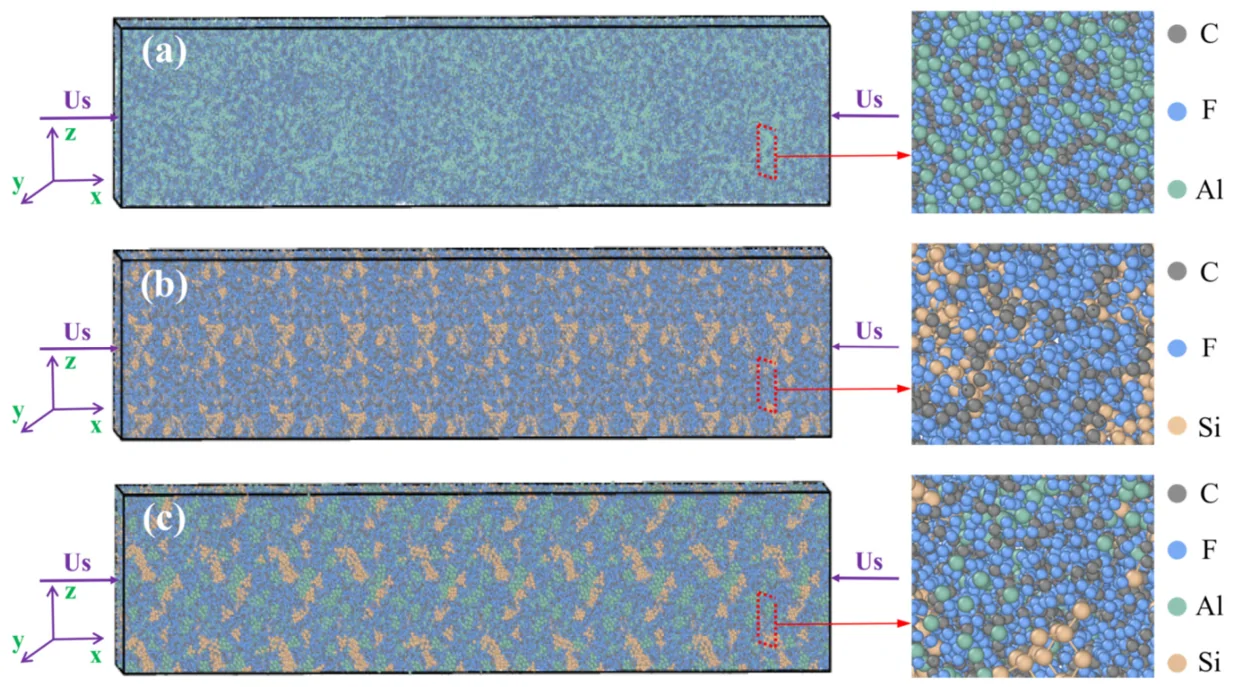
Initial models of RMs and Multiscale Shock Technique loading: (**a**) Al/PTFE; (**b**) Si/PTFE; (**c**) Al_1.2_Si_1_PTFE.

To mitigate the size effect on simulation results, the model dimensions for all Al_1.2_Si_1_PTFE RMs were set to 50 nm × 10 nm × 10 nm. In all simulations, the reactive materials were prepared with mass ratios of Al/PTFE (26.5/73.5), Si/PTFE (21.9/78.1), and Al_1.2_Si_1_PTFE (13.25/10.95/75.8). Their total atomic numbers are 368605, 355760, and 363600, respectively, and the initial densities are 2.237, 2.088, and 2.156 g/cm^3^. Each PTFE molecular chain consists of 64 tetrafluoroethylene molecules, with a polymerization degree of 128 [27]. The specific material types and distributions are shown in Figure 6. Grey spheres represent C atoms, and blue spheres represent F atoms (i.e., PTFE molecules). Green spheres represent Al atoms, and brown spheres represent Si atoms. It can be clearly seen that in all three RMs, Al and Si atoms are uniformly distributed within the PTFE molecular chains.

To ensure all atomic information can be exported, the “full” atom type was used, and the “real” unit was adopted in the simulation. The EAM [28] potential function was assigned to the Al element, the Tersoff [29] potential to the Si element, and the Lennard–Jones (LJ) [30] potential to interactions involving PTFE and between elements. Corresponding potential functions were depicted in Equations (4)–(6), respectively.

(4) $$E_{E}=\sum_{i=1}^{N}F_{i}(\rho_{i})+\frac{1}{2}\sum_{i=1}^{N}\sum_{\begin{array}{c} j=1\\ j\neq i \end{array}}^{N}\Phi_{\mathrm{ij}}(r_{\mathrm{ij}})$$

where *E*_E_ represents the overall energy of the system, including the self-embedding energy of atoms *F_i_*(*ρ_i_*) and the pairwise interaction energy between two atoms *Φ_ij_*(*r_ij_*). *ρ_i_* stands for the local electron density of atom *i* formed by the superposition of the electron densities generated by all surrounding neighboring atoms *j* at the position of atom *i*. *r_ij_* represents the interatomic distance.

(5) $$E_{T}=\frac{1}{2}\sum_{i}\sum_{i\neq j}{\;f}_{c}(r_{\mathrm{ij}})[f_{R}(r_{\mathrm{ij}})+b_{\mathrm{ij}}f_{A}(r_{\mathrm{ij}})]$$

Here, *E*_T_ is the overall energy of the system, including a two-body repulsive term *f*_R_(*r_ij_*) and three-body attractive interactions *f*_A_(*r_ij_*). *f*_C_(*r_ij_*) stands for the cutoff function. *b_ij_* represents the bond order parameter.

(6) $$V_{\mathrm{LJ}}(r_{\mathrm{ij}})=4\varepsilon_{\mathrm{ij}}[{\left(\frac{\sigma_{\mathrm{ij}}}{r_{\mathrm{ij}}}\right)}^{12}-{\left(\frac{\sigma_{\mathrm{ij}}}{r_{\mathrm{ij}}}\right)}^{6}]$$

where *V*_LJ_(*r_ij_*) denotes the L-J interaction energy between atom *i* and atom *j*. *ε_ij_* is the well depth, reflecting the strength of interatomic interactions. *σ_ij_* is the equilibrium distance, corresponding to the interatomic distance when *V*_LJ_(*r_ij_*) = 0.

Period boundary conditions were set in the x, y, and z directions. The conjugate gradient (CG) method was applied to minimize the energy of the initial model to eliminate unreasonable stresses in the initial configuration and optimize atomic arrangements to provide a stable starting state for subsequent dynamic simulations. A Langevin thermostat was used to relax the Al/PTFE, Si/PTFE, and Al_1.2_Si_1_PTFE models for 5 ps to achieve reasonable initial structures. Both the starting and target temperatures were set to 300.0 K (room temperature), and the damping time was set to 100 simulation timesteps. During this 5ps relaxation, the thermostat maintained constant-temperature conditions to equilibrate atomic configurations, and this Langevin fix was deactivated before the subsequent MSST shock compression simulation. Finally, the MSST method was employed at ambient temperature to apply the same impact velocity *U*_S_(2 km/s) in the x-direction for 5 ps.

### 3.2. Model Validation

To verify the accuracy of the potential functions, the MSST method was first used to perform the shock compression on Al, Si, and PTFE elemental materials in the x-direction. The experimental data for Al and PTFE were obtained from the Los Alamos Scientific Laboratory(LASL) experimental database provided by Marsh et al. [31] and those for Si were from Stefan et al. [32] and Strickson et al. [33].

**Figure 7:**
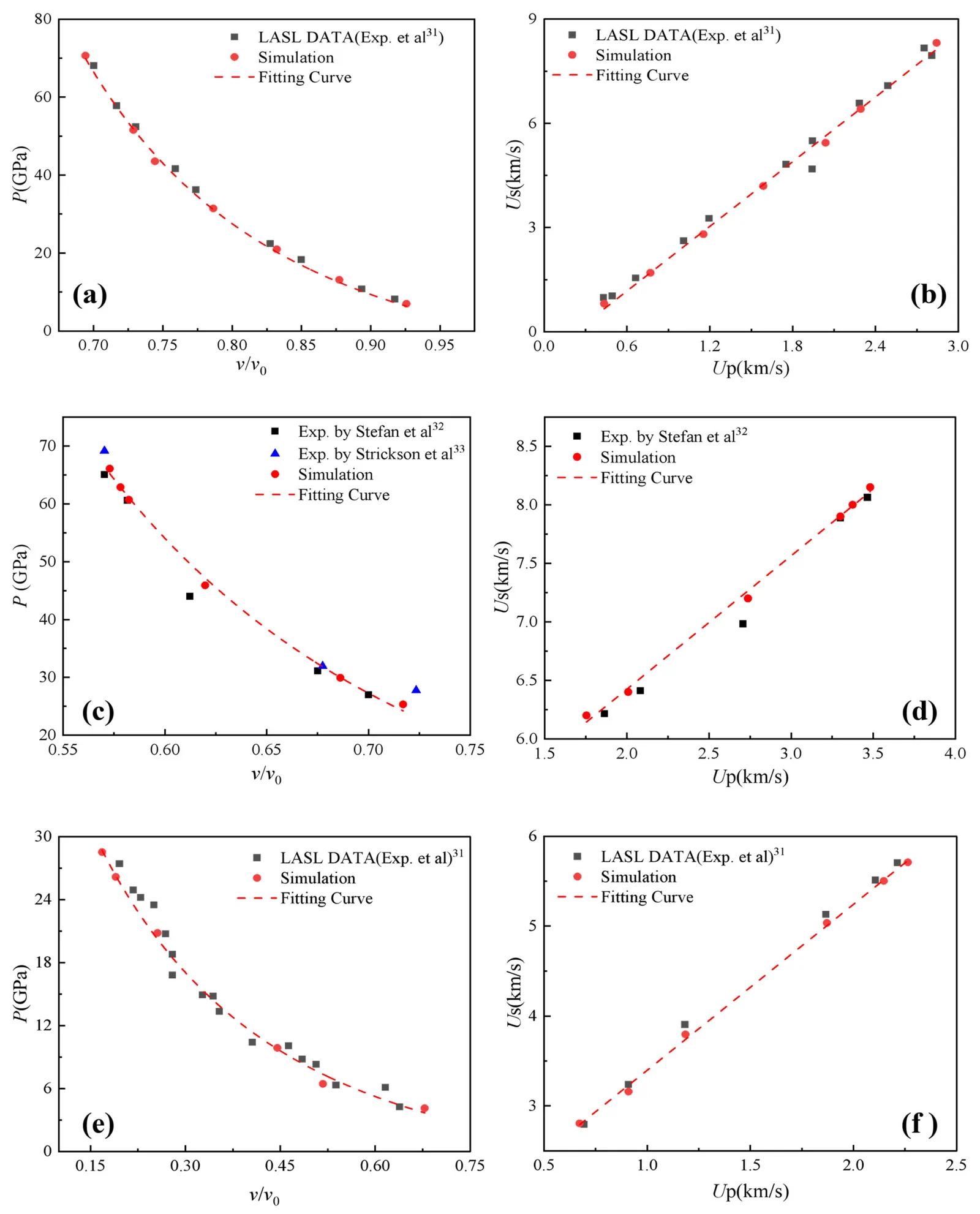
Shock Hugoniot relations of Al, Si, and PTFE elements (**a**) *P* − *v*/*v*_0_ for Al; (**b**) *U*s − *U*p for Al; (**c**) *P* − *v*/*v*_0_ for Si; (**d**) *U*s − *U*p for Si; (**e**) *P* − *v*/*v*_0_ for PTFE; (**f**) *U*s − *U*p for PTFE.

The comparative Hugoniot relationships between the simulation and experiment are shown in Figure 7, where the red dots indicate simulation results. The experimental data points are found to closely align with the *P* − *v*/*v*_0_ and *U*s − *U*p relationships derived from the simulation data (represented by the red dashed lines). The coefficients of determination R^2^ of the fitted curves for Al, Si, and PTFE are 0.972, 0.982, and 0.966, respectively.

This high correlation coefficient demonstrates the excellent fit between the data points and the fitted curves, thereby confirming the reliability of the potential functions used in the simulation without chemical reaction.

## 4. Results and Discussion

### 4.1. Impact Ignition Analysis

**Figure 8:**
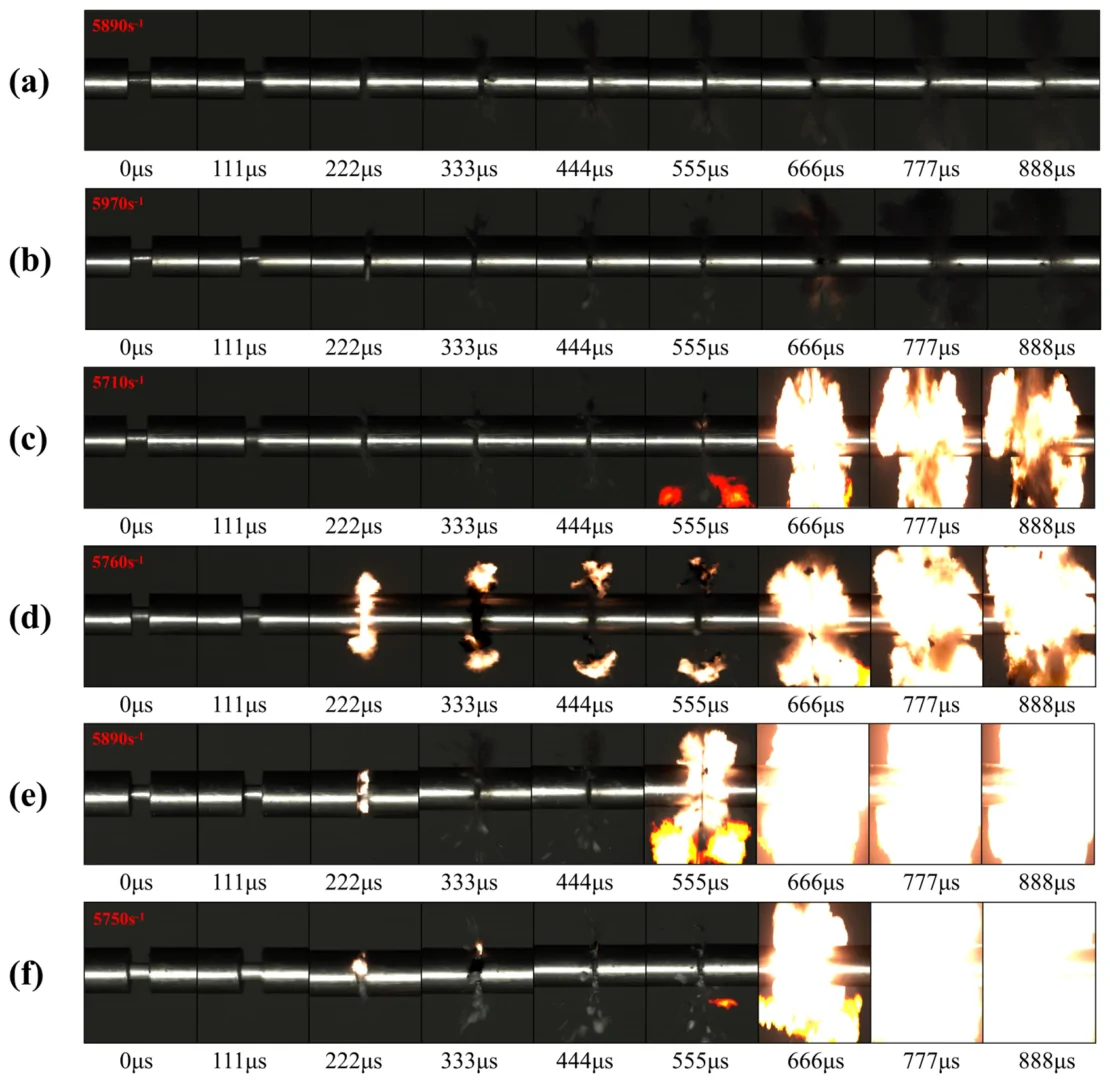
Comparisons of ignition characteristics of Al/Si/PTFE under different Al/Si mass ratios: (**a**) Si/PTFE; (**b**) Al/Si(0.33:1); (**c**) Al/Si(0.5:1); (**d**) Al/Si(1:1); (**e**) Al/Si(2:1); (**f**) Al/PTFE.

The delayed impact ignition characteristics of Al/Si/PTFE ternary RMs under strain rates ranging from 5710 s^−1^ to 5970 s^−1^ are illustrated in Figure 8. It can be seen from Figure 8a that the Si/PTFE (Al: Si = 0:1) only undergoes a compression deformation and fragmentation under a single compressive pulse at a strain rate of 5890 s^−1^, without ignition reaction. After adding a small amount of Al powder (Al: Si = 0.33:1), it can be observed that the Al/Si/PTFE ternary energetic material produces scattered sparks radiating outward at 666 μs, indicating partial ignition during the second loading (Figure 8b). Thus, adding Al powder reduces the reaction threshold of Si/PTFE RMs. As Al content gradually increases, the Al/Si/PTFE ternary RMs exhibit more intense shock ignition (Figure 8c–f).

Compared to the shock ignition of Al/PTFE (Figure 8f), when the mass ratio of Al: Si is smaller than 0.5:1 (Figure 8b,c), Al/Si/PTFE RMs will not be ignited during the first loading. This occurs due to two reasons: firstly, the high Si content in Al/Si/PTFE improves the plasticity of Al/PTFE, which fails to generate sufficient energy to trigger ignition. Secondly, Si disrupts the orderly arrangement of Al particle networks in PTFE, reducing local stress concentrations and releasing insufficient energy to reach the ignition state. When the mass ratio Al: Si is ≥1:1 (Figure 8d–f), Al/Si/PTFE ternary RMs will be ignited during the first loading at 222 μs as Al content increases. Notably, at a mass ratio of Al: Si = 1:1 (Figure 8d), Al/Si/PTFE undergoes intense and sustained reactions after the first ignition. With further increases in Al content (Figure 8e,f), the first ignition does not trigger sustained chemical reactions; instead, intense reactions occur under the second compressive pulse. These experimental results indicate that at high strain rates (around 5800 s^−1^), Al/Si/PTFE with a mass ratio near 1:1 has a lower reaction threshold and more intense energy release than Al/PTFE.

**Figure 9:**
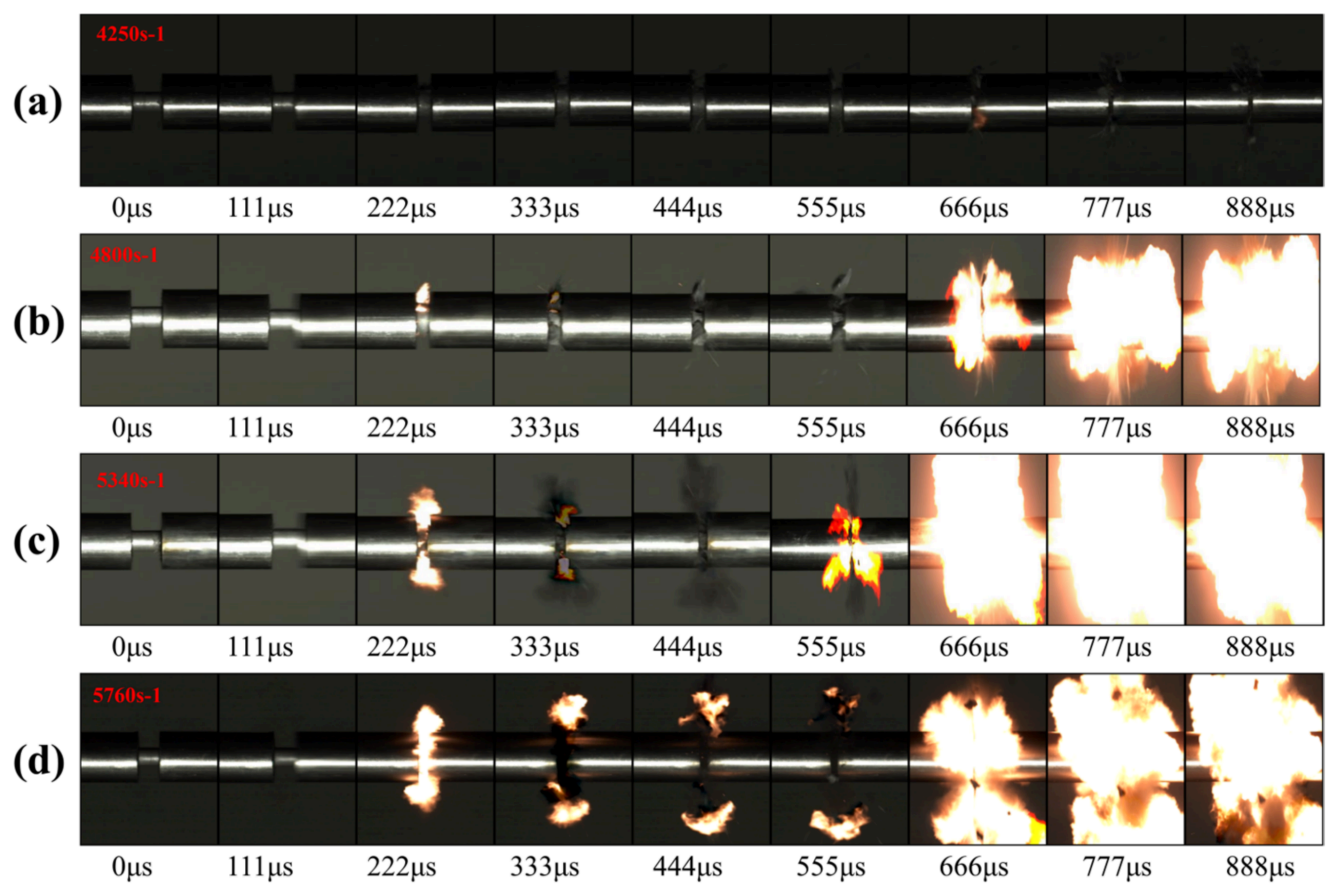
Shock ignition reactions and energy release behavior of Al_1.2_Si_1_PTFE under different strain rates (**a**) 4250 s^−1^; (**b**) 4800 s^−1^; (**c**) 5340 s^−1^; (**d**) 5760 s^−1^.

Therefore, the delayed ignition reaction characteristics of Al_1.2_Si_1_PTFE under different strain rates are compared and shown in Figure 9. At a strain rate of 4250 s^−1^, Al_1.2_Si_1_PTFE only shows faint ignition at 666 μs (within the second loading). A higher strain rate of 4800 s^−1^ reveals clear ignition during the first pulse (222 μs), but this ignition does not trigger sustained reactions. Instead, significant reaction behavior with intense light occurs during the second loading phase (666–777 μs). When strain rates are ≥5340 s^−1^, Al_1.2_Si_1_PTFE will be ignited at both 222 μs and 666 μs, and the reaction will sustain after the first ignition at higher strain rates. Compared to shock ignition under a single compressive pulse, the ignition reaction under the second compressive pulse is more intense and prolonged. In summary, the shock ignition and intense energy release behavior of Al_1.2_Si_1_PTFE are jointly affected by the Al/Si mass ratio and strain rate.

### 4.2. Impact Reaction Analysis

**Figure 10:**
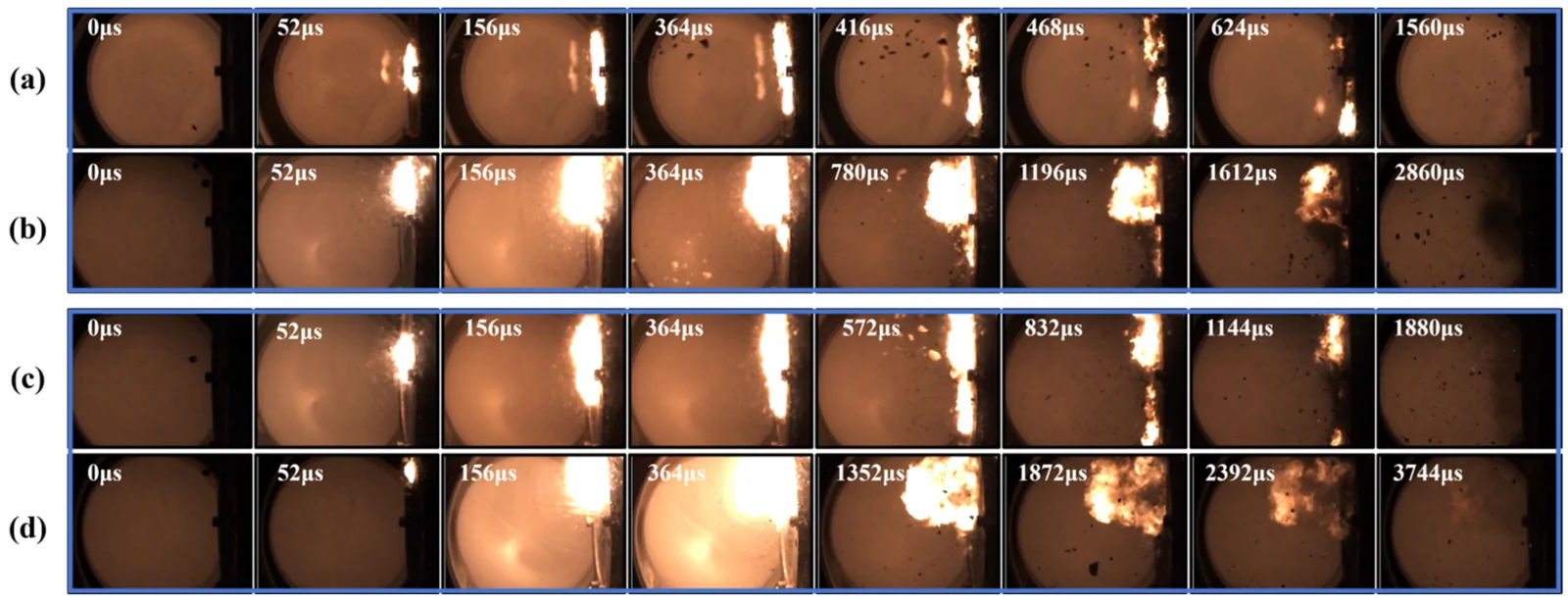
Comparison of reaction characteristics of Al/PTFE and Al_1.2_Si_1_PTFE at different impact velocities (**a**) Al/PTFE(650 m/s) (**b**) Al_1.2_Si_1_PTFE(650 m/s) (**c**) Al/PTFE(750 m/s) (**d**) Al_1.2_Si_1_PTFE(750 m/s).

The comparison of reaction energy release between Al/PTFE and Al_1.2_Si_1_PTFE under different impact velocities is shown in Figure 10. By comparing Figure 10a with Figure 10b, and Figure 10c with Figure 10d, it can be concluded that the impact energy release performance of the Al_1.2_Si_1_PTFEternary composite is significantly superior to that of the Al/PTFE binary composite at impact velocities of 650 m/s and 750 m/s. Al_1.2_Si_1_PTFE exhibits a longer reaction duration than Al/PTFE with a gentle extinction process rather than sudden quenching. Such enhancement in reaction duration becomes more prominent as the impact velocity increases.

**Figure 11:**
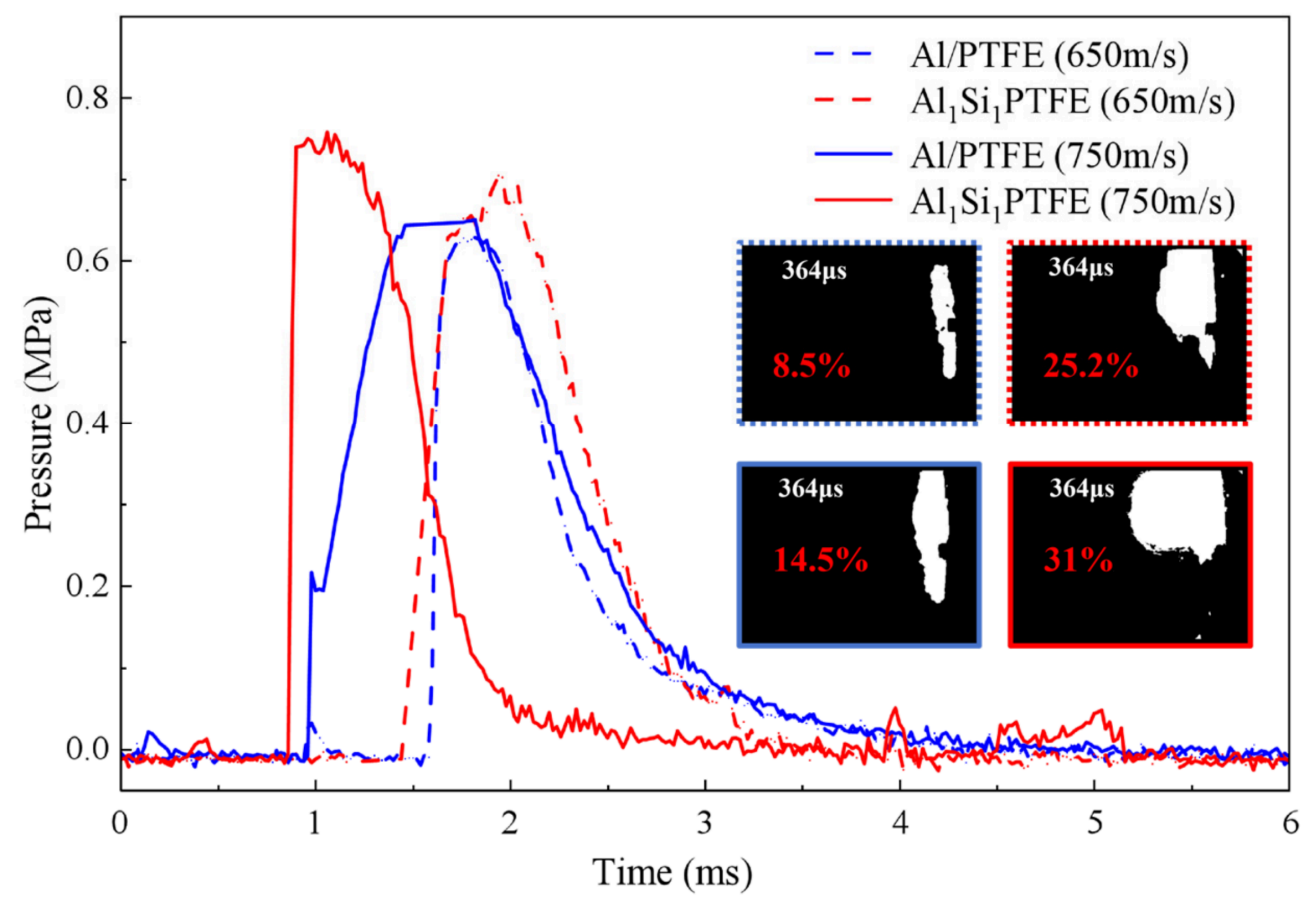
Pressure histories of Al/PTFE and Al_1.2_Si_1_PTFE at different impact velocities.

Figure 11 shows the pressure histories of Al/PTFE and Al_1.2_Si_1_PTFE at different impact velocities. The peak pressures of Al_1.2_Si_1_PTFE are higher than those of Al/PTFE, indicating more energy release. The flame occupancy ratio of material reactions at 364 μs is also shown in Figure 11, with the blue and red curves illustrating the time-dependent pressure variations in Al/PTFE and Al_1.2_Si_1_PTFE, respectively, where the dashed lines and solid lines denote the pressure curves at 650 m/s and 750 m/s, respectively. During the reaction, the sparks generated by Al_1.2_Si_1_PTFE exhibit an approximate spherical-cylindrical morphology. Compared with the flat sparks of Al/PTFE, Al_1.2_Si_1_PTFE possesses a wider diffusion range with uniform brightness. 

The peak pressure and reaction duration of the two RMs at two impact velocities are summarized in Table 2. It can be observed that the peak pressure of Al_1.2_Si_1_PTFE is approximately 12% higher than that of Al/PTFE at the impact velocity of 650 m/s. At 750 m/s, the peak pressure of Al_1.2_Si_1_PTFE increases by about 16.5% compared with Al/PTFE.

To further compare the reaction efficiency of the two RMs, the chemical energy obtained from the experiment is compared with the chemical energy produced by the complete reaction of the materials. The equations for the complete reaction of Al/PTFE and Si/PTFE at zero oxygen ratio are as follows:

4Al + 3C_2_F_4_ = 4AlF_3_ + 6C          ΔHmass = −5.82 kJ/g (7)

Si + C_2_F_4_ = SiF_4_ + 2C            ΔHmass = −6.21 kJ/g (8)

C + O_2_ = CO_2_               ΔHmass = −5.74 kJ/g (9)

Thus, the chemical energy per unit mass of Al/PTFE and Al_1.2_Si_1_PTFE is 11.564 kJ/g and 11.759 kJ/g, respectively. Assuming that the combustion chamber is a closed system in the few microseconds before the quasi-static pressure reaches its peak, the relationship between the peak value and the energy increment inside the combustion chamber is as follows.

(10) $$\Delta P=\frac{\gamma -1}{V}\Delta Q$$

Here, Δ*P* is the quasi-static pressure peak (MPa), and *V* is the effective volume of the quasi-sealed container (L). Considering the small size of the reactive material (RMs), the high-pressure gas generated by combustion cannot fill the entire closed cavity. In this study, the space enclosed from the explosion center to the pressure sensor measuring point is defined as the effective combustion volume, and the effective volume is taken as 1.07 L. Δ*Q* is the energy increment inside the quasi-sealed container (kJ), including kinetic and chemical energy, and *γ* = 1.4 is the specific heat ratio of air. 

The chemical energy and reaction efficiency of Al/Si/PTFE RMs are listed in Table 2. Results indicate that the addition of a certain amount of Al to Si/PTFE can significantly enhance the reaction rate and extent of Si/PTFE. Since materials with higher reaction extents react faster under the same pressure, the reaction extent and rate of Al_1.2_Si_1_PTFE are both higher than those of Al/PTFE. 

### 4.3. Microscopic Mechanism

**Figure 12:**
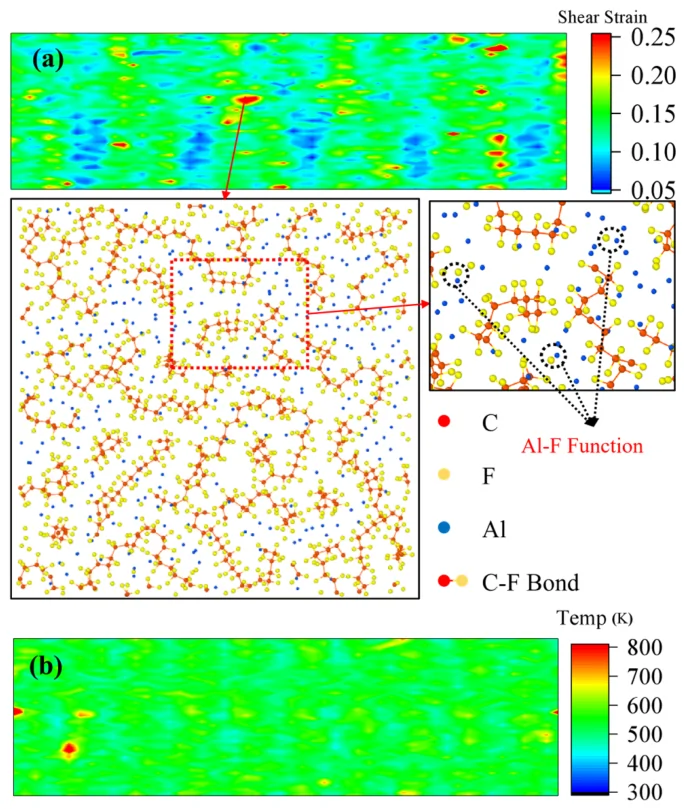
Thermodynamic response and microstructural changes of Al/PTFE (1 ps): (**a**) shear strain distribution and local microstructure; (**b**) temperature contour map.

**Figure 13:**
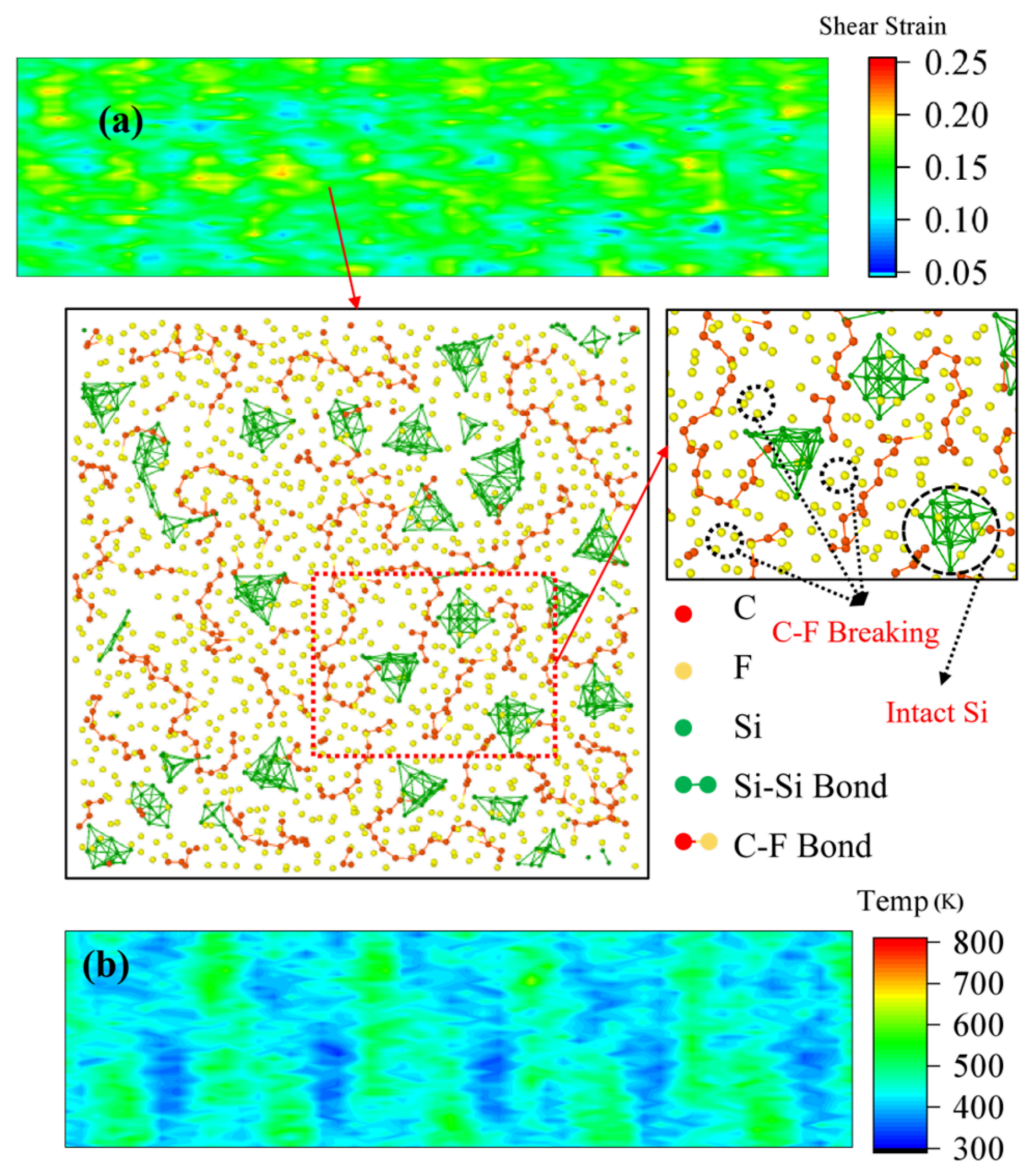
Thermodynamic response and microstructural changes of Si/PTFE (1 ps): (**a**) shear strain distribution and local microstructure; (**b**) temperature contour map.

**Figure 14:**
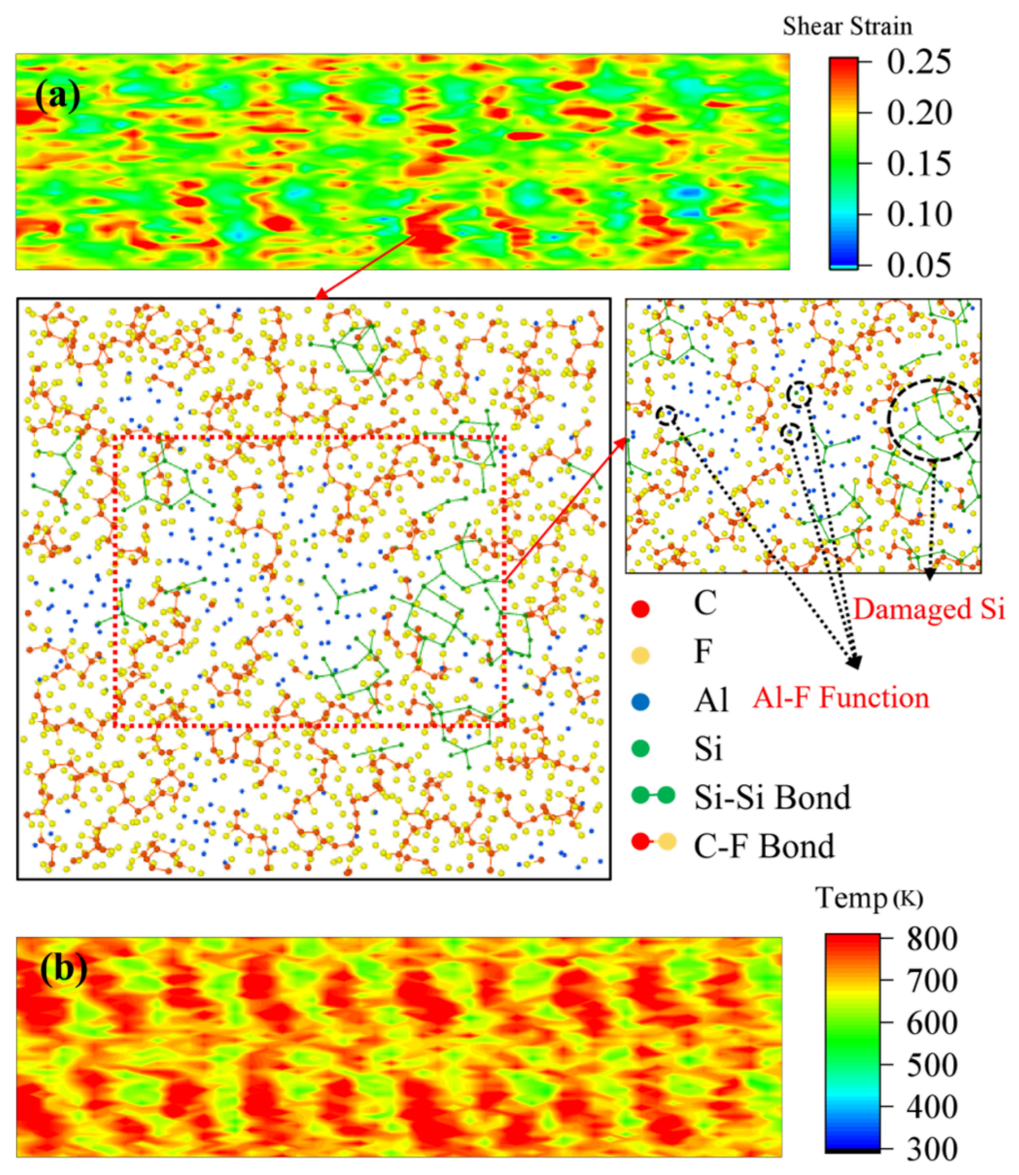
Thermodynamic response and microstructural changes of Al_1.2_Si_1_PTFE (1 ps): (**a**) shear strain distribution and local microstructure; (**b**) temperature contour map.

Based on the above experimental results, the MSST method is utilized to perform shock loading in the x-direction on three RMs: Al/PTFE, Si/PTFE, and Al_1.2_Si_1_PTFE, respectively. The shear strain contours, local microstructures of high-strain regions, and temperature contours of Al/PTFE, Si/PTFE, and Al_1.2_Si_1_PTFE at 1 ps are presented in Figure 12, Figure 13 and Figure 14, respectively. 

By comparing Figure 12a, Figure 13a and Figure 14a, it can be observed that the high shear strain regions of the Al_1.2_Si_1_PTFE are significantly more extensive than those of Al/PTFE and Si/PTFE. This is due to two reasons: on one hand, the high hardness and strength of Si hinder the plastic flow of Al, forcing Al to undergo local shear sliding around Si particles, which promotes the formation and cross-expansion of numerous local shear bands, macroscopically manifesting as more significant shear deformation. On the other hand, the micro-pyramid structure on the Si surface causes a high local concentration of shock energy, making the C-F bonds in PTFE subjected to more intensive tensile or shear forces, leading to the breakage of a large number of C-F bonds and the release of F atoms, as shown in the microstructure diagram of Si/PTFE in Figure 12a. Therefore, in Al_1.2_Si_1_PTFE, a large number of F atoms interact with Al atoms, releasing more energy compared with that in Al/PTFE. Under the coupled effect of shock and aluminum–fluorine bonding, Si is damaged, as shown in the microstructure of Si atoms in Figure 13a. The deformation and fracture of Si can increase the interface temperature, which in turn promotes the thermal decomposition of PTFE, and the decomposition of PTFE will conversely intensify the interaction between Al and F, thereby forming a positive cycle and further enhancing the energy release of the material. Thus, the internal temperature rise of Al_1.2_Si_1_PTFE under high-speed shock is significantly higher than that of Al/PTFE and Si/PTFE, resulting in more hotspots that trigger ignition (Figure 12b, Figure 13b and Figure 14b).

By comparing Figure 12a, Figure 13a and Figure 14a, it can be seen that the high shear strain regions of the Al_1.2_Si_1_PTFE system are significantly larger than those of the Al/PTFE and Si/PTFE systems. The local temperature rise induced by shock deformation exceeds the thermal reaction threshold of the composite in the microscopic regions shown in the figures, so bond breakage and chemical reactions preferentially occur in such high-temperature zones. The high-hardness silicon particles hinder the plastic flow of aluminum and induce intense localized shear deformation and local temperature elevation. Combined with the critical reaction temperature of the material obtained from DSC tests (Section 2.2: Al/PTFE-803.15 K, Si/PTFE-843.15 K, Al_1.2_Si_1_PTFE-793.15 K), the local high temperature (< 800 K) is still below the critical reaction temperature of the PTFE-based composites. However, it is suggested that these atomic positions are about to form hotspots, eventually reaching critical temperatures for chemical reactions such as bond breaking and formation, as shown in Figure 12a, Figure 13a and Figure 14a.

Under the effect of intense shear deformation, chemical reactions are preferentially activated in Al_1.2_Si_1_PTFE at the positions indicated in the figures. During the reaction, the PTFE matrix undergoes thermal decomposition, resulting in temperature rise and forming a favorable reaction cycle. Consequently, the overall temperature rise of Al_1.2_Si_1_PTFE under high-speed impact is much higher than that of Al/PTFE and Si/PTFE, generating more high-temperature hotspots and ultimately making the material easier to ignite.

**Figure 15:**
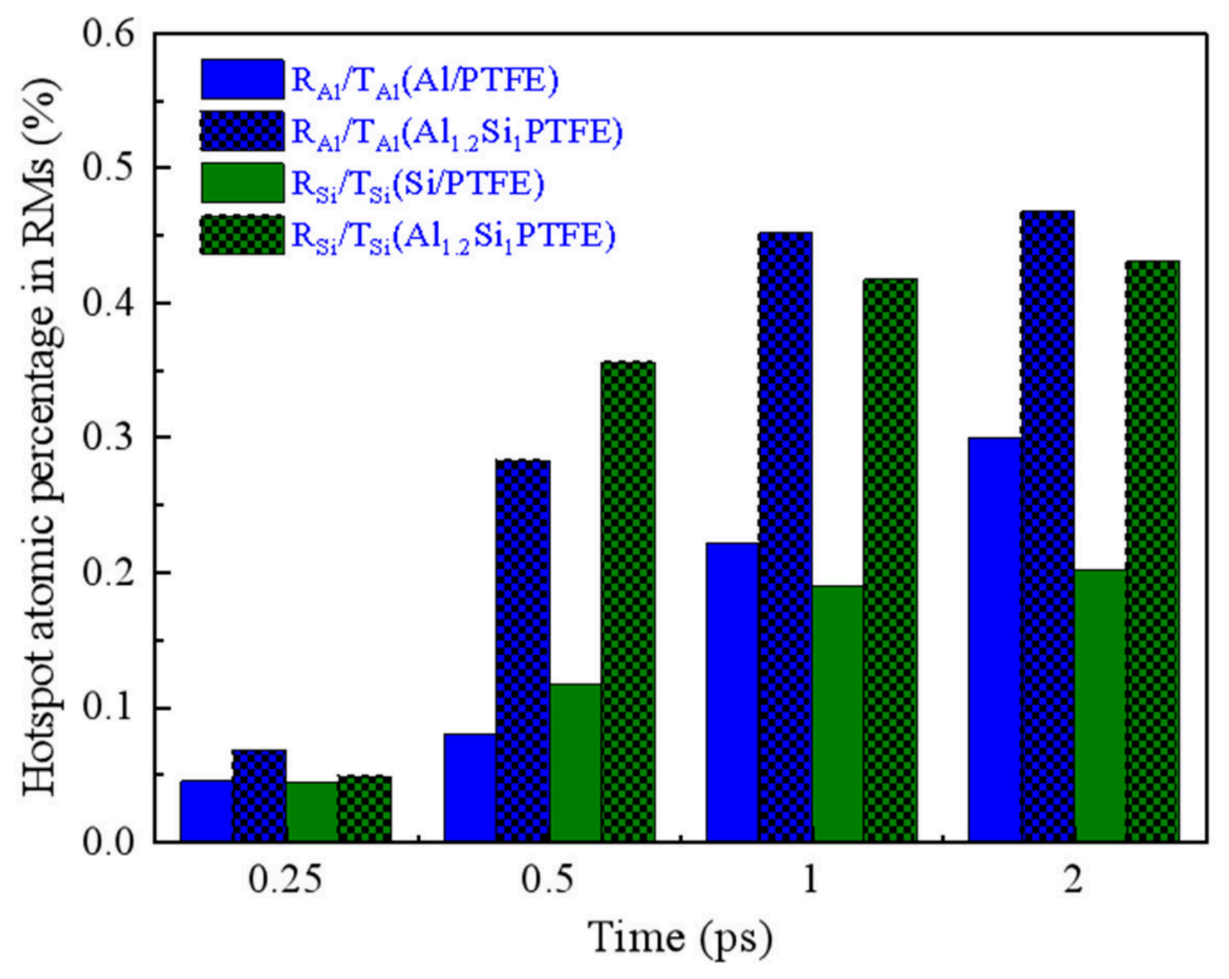
Reaction proportion diagram of reductive particles (Al, Si) in Al/PTFE, Si/PTFE, and Al_1.2_Si_1_PTFE.

In addition, according to the DSC test results in Figure 4, the critical reaction temperature thresholds of Al/PTFE and Si/PTFE are 530 °C and 570 °C, respectively, which can be used as the basis for simulating the reactions of Al and Si atoms. Figure 15 compares the hotspot atomic percentage of Al/PTFE, Si/PTFE, and Al_1.2_Si_1_PTFE RMs at different times. The ordinate denotes the proportion of Al and Si atoms forming hotspots, which is calculated based on the initial total atomic number of each element in the reactive materials (RMs) of Al/PTFE, Si/PTFE, and Al_1.2_Si_1_PTFE. For instance, R_Al_/T_Al_(Al/PTFE) represents the proportion of Al atoms reaching the hotspot temperature in Al/PTFE RMs; R_Si_/T_Si_(Si/PTFE) stands for the proportion of Si atoms reaching the hotspot temperature in Si/PTFE. The bar charts corresponding to R_Al_/T_Al_(Al/Si/PTFE) and R_Si_/T_Si_(Al/Si/PTFE) indicate the proportions of Al and Si atoms reaching the hotspot temperature in the Al_1.2_Si_1_PTFE system, respectively. It can be clearly observed that the number of hotspots in Al_1.2_Si_1_PTFE under shock wave loading is markedly higher than those in Al/PTFE and Si/PTFE. Therefore, from the perspective of microscopic hotspot formation, Al_1.2_Si_1_PTFE exhibits more sufficient energy release under shock loading compared with Al/PTFE and Si/PTFE, which is consistent with the experimental phenomena.

## 5. Conclusions

Previous studies on Al/Si/PTFE have largely been confined to binary systems and strategies for adding trace amounts of third elements, lacking systematic exploration of Al-Si synergism and the underlying microscale shock ignition mechanism in ternary composites [34]. In this work, Al/Si/PTFE mixtures with varied mass ratios were fabricated and characterized via SEM/EDS, DSC and SHPB, while molecular dynamics simulations were adopted to uncover the ignition mechanism of Al_1.2_Si_1_PTFE.

The Al/Si/PTFE RMs of various mass ratios were prepared and characterized by SEM/EDS. The shock-induced ignition delay and energy-release behaviour of Al/Si/PTFE RMs were examined via SHPB and DSC tests, respectively. MD simulations were performed to reveal the micro-scale ignition mechanism of Al_1.2_Si_1_PTFE. The addition of Al powder can effectively reduce the reaction threshold and improve the energy release efficiency of non-metallic Si/PTFE. The Al_1.2_Si_1_PTFE underwent intense primary and secondary ignition within the range of 5710 S^−1^–5970 S^−1^. The shock-induced ignition delay was shortened, and the reaction of Al_1.2_Si_1_PTFE was most vigorous, demonstrating that it possesses the maximum energy release efficiency. Al/PTFE and Si/PTFE began to undergo exothermic reaction at 530 °C and 570 °C, respectively, whereas Al_1.2_Si_1_PTFE did so at 520 °C, indicating that the addition of Al lowers the critical reaction-temperature threshold. When subjected to dynamic shock, the soft Al in Al_1.2_Si_1_PTFE was intensely sheared against the hard Si, which increases local strain and temperature. This shear promotes Al–F contact and reaction. Consequently, more hotspots form within Al_1.2_Si_1_PTFE, facilitating ignition. According to the proportion of reactive atoms, Al_1.2_Si_1_PTFE reacted significantly more extensively than either Al/PTFE or Si/PTFE.

## Figures and Tables

**Table 1:** Physical properties of Al/Si/PTFE RMs

Sample	Mass Ratio of Al to Si	Mass Ratio	Density g/cm^3^	TMD g/cm^3^	Relative Density/%
1#	Al/Si/PTFE (0:1)	0/21.9/78.1	2.088	2.170	96.2
2#	Al/Si/PTFE (0.33:1)	5.68/17.21/77.2	2.139	2.191	97.6
3#	Al/Si/PTFE (0.5:1)	7.74/15.50/76.76	2.142	2.199	97.4
4#	Al/Si/PTFE (1.2:1)	13.25/10.95/75.8	2.156	2.220	97.1
5#	Al/Si/PTFE (2:1)	16.51/8.26/75.23	2.157	2.233	96.6
6#	Al/Si/PTFE (1:0)	26.5/0/73.5	2.237	2.273	98.4

**Table 2:** Comparison of reaction efficiency of two types of materials.

Samples	Mass	Velocity	Time	P_max_	Experiment Energy (kJ)			Theory Energy (kJ)	Reaction Efficiency
					Total	Kinetic	Chem.		
Al/PTFE	0.22	650	1560	0.626	1.68	0.05	1.63	2.54	64.3
		750	1880	0.650	1.74	0.06	1.68	2.54	66.2
Al_1.2_Si_1_PTFE	0.20	650	2860	0.704	1.89	0.04	1.85	2.35	78.5
		750	3744	0.758	2.04	0.06	1.98	2.35	84.1

## Data Availability

The original contributions presented in this study are included in the article/Supplementary Materials. Further inquiries can be directed to the corresponding authors.

## Supplementary Materials

The following supporting information can be downloaded at: https://www.mdpi.com/article/10.3390/polym18192432/s1, Figure S1: Pulsed voltage signal and reflected strain diagrams of the Al_1.2_Si_1_PTFE material; Figure S2: Engineering strain diagram of Al_1.2_Si_1_PTFE materials. Refs. [35,36] were cited in the Supplementary Materials.
