# Supplementary material for: Atomic-Scale Reaction Mechanism of Impact Ignition in Al/Si/PTFE Reactive Materials
Source: Polymers (Basel). 2026 Oct 6;18(19):2432. doi: 10.3390/polym18192432 (PMC13667215; doi:10.3390/polym18192432)
Supplement: Supplementary file 1 [file polymers-18-02432-s001.zip › polymers-4581353-supplementary.pdf]

Supplementary materials.

Atomic-scale reaction mechanism of impact ignition in Al/Si/PTFE reactive materials

Liangfei Gong [1], Lieke Ma [1], Xiaomalong Pu [1], Bo Li [1], Yulian Liu [1], Ao Mei [1,*] and Jie Wang [2,*]

[1]State Key Laboratory of Mountain Bridge and Tunnel Engineering, Chongqing Jiaotong Uni-versity, Chongqing 400074, China

[2]College of Aerospace Engineering, Chongqing University, Chongqing 400044, China

[*]Correspondence: meiao@cqjtu.edu.cn (A.M.); jiewang_cae@cqu.edu.cn (J.W.):

1 SHPB methodology

According to the theory of the SHPB experimental technique, the average stress, strain, and strain rate of the specimen under dynamic loading can be obtained using the following relationships:

$$\sigma(t) = \frac{EA_0}{2A_S}[\varepsilon_I(t) + \varepsilon_R(t) + \varepsilon_T(t)] \tag{S.1}$$

$$\varepsilon(t) = \frac{C_0}{L_S}\int_0^t [\varepsilon_I(t) - \varepsilon_R(t) - \varepsilon_T(t)]\, dt \tag{S.2}$$

$$\dot{\varepsilon}(t) = \frac{C_0}{L_S}[\varepsilon_I(t) - \varepsilon_R(t) - \varepsilon_T(t)] \tag{S.3}$$

where, A_0 is defined as the cross-sectional area of the pressure bar, A_s is defined as the cross-sectional area of the specimen, L_s ,is defined as the initial length of the specimen, and $\varepsilon_I(t)$, $\varepsilon_R(t)$ and $\varepsilon_T(t)$ are defined as the incident strain, reflected strain, and transmitted strain, respectively.

Based on the one-dimensional stress wave theory, **Eqs. (S.1) - (S.3)** can be simplified as follows:

$$\sigma(t) = \frac{EA_0}{2A_S}\varepsilon_T(t) \tag{S.4}$$

$$\varepsilon(t) = \frac{2C_0}{L_S}\int_0^t \varepsilon_R(t) dt \tag{S.5}$$

$$\dot{\varepsilon}(t) = \frac{2C_0}{L_S}\varepsilon_R(t) \tag{S.6}$$

The following relationship is established between the pulsed voltage signal and the reflected strain:

$$\varepsilon_R(t) = \frac{U_{out}}{KU_{in}} \tag{S.7}$$

here, K is taken as the sensitivity coefficient with a value of 0.3, U_{in} is the input voltage with a value of 2.22, and U_{out} is the output voltage. The pulsed voltage signals and reflected strains for materials #1, #6, and #8 are shown in **Fig. S1**.

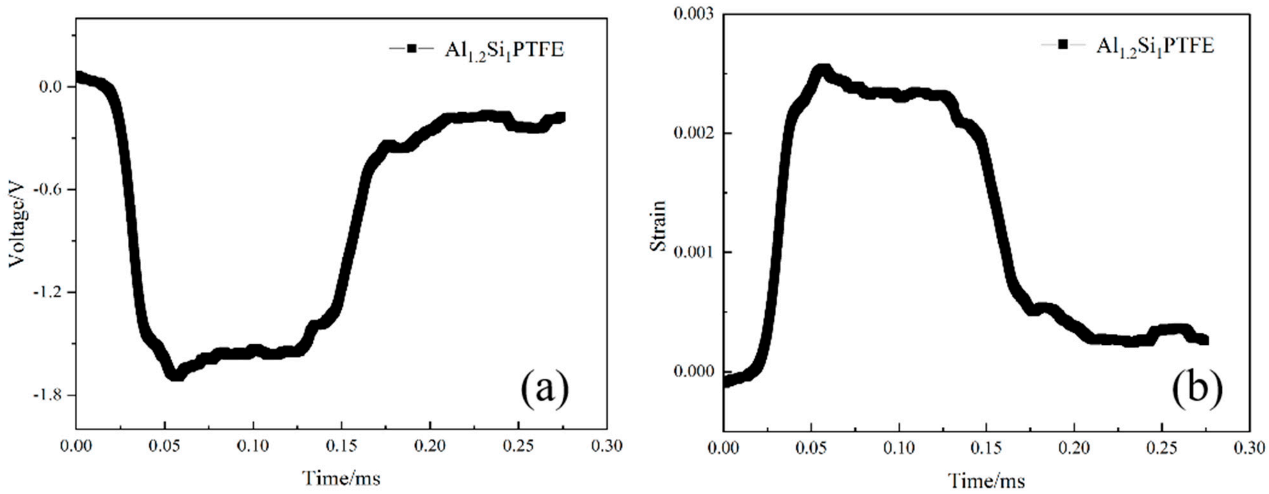

Figure S1 Pulsed voltage signal and reflected strain diagrams of the $Al_{1.2}Si_1PTFE$ material: (a) pulsed voltage signal, (b) reflected strain

Based on **Eq. (S.5),** the engineering strain of the material is obtained, as shown in **Fig. S2.** The slope of the strain curve is defined as the strain rate of the material.

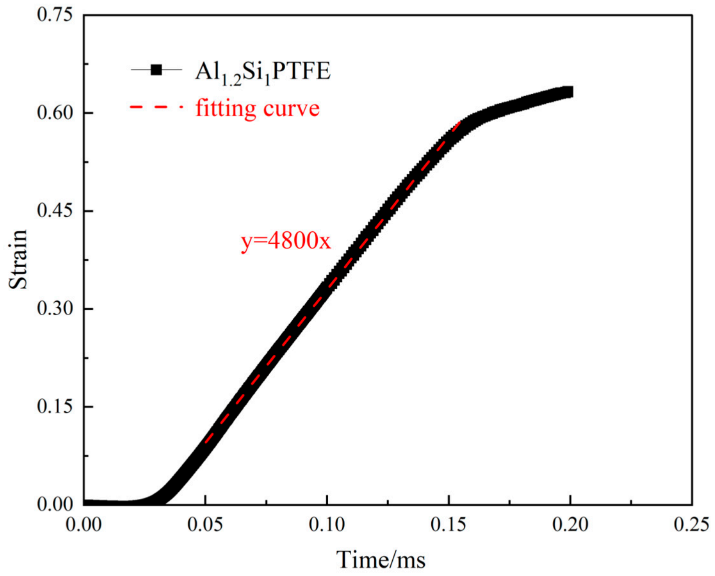

Figure S2 Engineering strain diagram of $Al_{1.2}Si_1PTFE$ materials

2 Thermochemical calculations

This paper has clearly defined the ΔH value based on mass, with specific calculations using $4Al+3C_2F_4=4AlF_3+6C$ → ΔH=-5.82 KJ/g as a reference,

**Element**	**State of matter**	**Δf H298° (kJ/mol)**	**Reference**
Al	solid	0.00	NIST-JANAF
C_2F_4	solid	-830.20[1]	ATcTActive
AlF_3	gas	-1209.32[2]	NIST-CCCBDB
C	solid(graphite)	0.00	NIST-JANAF

Then,

$$\Delta H = [4\Delta H(AlF3(g) + 6\Delta H(C(s)] - [4\Delta H(Al(s)) + 3\Delta(C2F4(s)]$$
$$= [4 \times (-1209.32) + 0] - [0 + 3 \times (-830.20)]$$
$$= -4837.28 + 2490.60$$
$$= -2346.68 \text{ kJ}$$

Total mass of the entire set of reactants: $m_{total} = 4\times 26.98 + 3 \text{ x } 100.02 = 407.98$ g

Unit mass change of pure chemical reaction at 298K: ΔHmass=-2346.68/407.98≈ -5.8kJ/g

[1] Ruscic, R. E. Pinzon, M. L. Morton, G. von Laszewski, S. Bittner, S. G. Nijsure, K. A. Amin, M. Minkoff, and A. F. Wagner, Introduction to Active Thermochemical Tables: Several "Key" Enthalpies of Formation Revisited. J. Phys. Chem. A 108, 9979-9997 (2004)

[2] Buchler A, Marram EP, and Stauffer JL. The Sublimation of Aluminum Trifluoride and the Infrared Spectrum of AlF3(g). J. Phys. Chem. Vol. 71 # 12. Pgs. 4139-414210.1021/j100871a072
